# Enhancing Mechanical Performance of a Polymer Material by Incorporating Pillar[5]arene-Based Host–Guest Interactions

**DOI:** 10.3390/gels8080475

**Published:** 2022-07-28

**Authors:** Chengdi Huang, Hanwei Zhang, Ziqing Hu, Youping Zhang, Xiaofan Ji

**Affiliations:** School of Chemistry and Chemical Engineering, Huazhong University of Science and Technology, Wuhan 430074, China; chengdihuang@hust.edu.cn (C.H.); zhanghanwei@hust.edu.cn (H.Z.); huziqingbjt@163.com (Z.H.); u201810425@hust.edu.cn (Y.Z.)

**Keywords:** polymer gels, host–guest interactions, mechanical performance, pillar[5]arene

## Abstract

Polymer gels have been widely used in the field for tissue engineering, sensing, and drug delivery due to their excellent biocompatibility, hydrophilicity, and degradability. However, common polymer gels are easily deformed on account of their relatively weak mechanical properties, thereby hindering their application fields, as well as shortening their service life. The incorporation of reversible non-covalent bonds is capable of improving the mechanical properties of polymer gels. Thus, here, a poly(methyl methacrylate) polymer network was prepared by introducing host–guest interactions between pillar[5]arene and pyridine cation. Owing to the incorporated host–guest interactions, the modified polymer gels exhibited extraordinary mechanical properties according to the results of the tensile tests. In addition, the influence of the host–guest interaction on the mechanical properties of the gels was also proved by rheological experiments and swelling experiments.

## 1. Introduction

Polymer gels, as an important material, have been widely applied in tissue engineering [1,2,3,4,5], sensing [6,7], and drug delivery [8,9], etc. However, in most cases, polymer gels are not endowed with enough mechanical strength, limiting their applications. The incorporation of physical crosslinkers in covalent polymer gels to construct a dual-crosslinked network is a desirable technique to enhance the mechanical properties of polymer gels [10]. Physical crosslinkers are based on reversible non-covalent bonds, which can dissipate vast quantities of energy through bond dissociation [11]. Due to this effective energy dissipation mechanism, polymer gels with physical crosslinkers can always bear a higher mechanical load, leading to outstanding toughness [12]. Apart from toughness, dual-crosslinked polymer gels are also capable of recovering their mechanical properties following relaxation, which is attributed to the cooperation of covalent crosslinking and the reversibility of non-covalent bonds [13,14,15]. Thus, incorporating physical crosslinkers in polymer gels is a promising strategy by which to improve the mechanical properties of polymer gels and has achieved much progress in numerous investigations [16].

To date, the most common physical crosslinkers include metal coordination [16,17], hydrogen bonds [18,19,20], and host–guest interactions [13,21,22,23,24]. Zhou et al. utilized Fe^3+^–acrylic acid coordination as the crosslink point to design a dual-crosslinked hydrogel network that exhibits outstanding toughness and mechanical performance [25]. Craig et al. developed a polymer gel network by incorporating bifunctional van Koten-type PINs as the reversible non-covalent bond [26]. This gel is endowed with excellent fracture stress, and a surprisingly short relaxation time was observed. Guan and co-workers surveyed the influence of hydrogen bonds on the mechanical properties of gels via incorporating secondary amide side chains in the gel network [15]. The results indicate a toughness over seven-fold stronger due to the dissociation of hydrogen bonds. A xerogel based on the host–guest interaction between β-cyclodextrin and adamantane was also reported [27]. This xerogel shows extraordinary tensile strength and great self-adhesive ability. Scherman and coworkers [14] constructed a dual-crosslinked network based on cucurbit[8]uril (CB[8])-mediated host–guest interactions, which endowed the polymer gels with excellent toughness, strength, elasticity, and recoverability. These studies evidence the significance of the introduction of physical crosslinkers that reinforce the mechanical performance of polymeric gels in many aspects [28,29]. Pillar[n]arenes [30,31,32,33], first introduced in 2008 [21], have been widely reported as important macrocyclic hosts due to their specific guest recognition [34,35,36,37,38,39,40], easily modifiable properties [33,40,41,42,43,44,45,46], and their rigid and symmetrical structure [33,47,48,49,50,51,52]. While the host–guest interactions based on pillar[n]arenes have been used for crosslinking linear polymers to obtain supramolecular polymer networks [53,54,55,56,57,58,59,60], few studies have focused on the influence of their host–guest interactions on the mechanical performance of covalent polymer gels. Thus, it is essential to develop a novel polymer gel incorporating host–guest interactions based on pillar[n]arenes [61,62,63].

**Figure 1:**
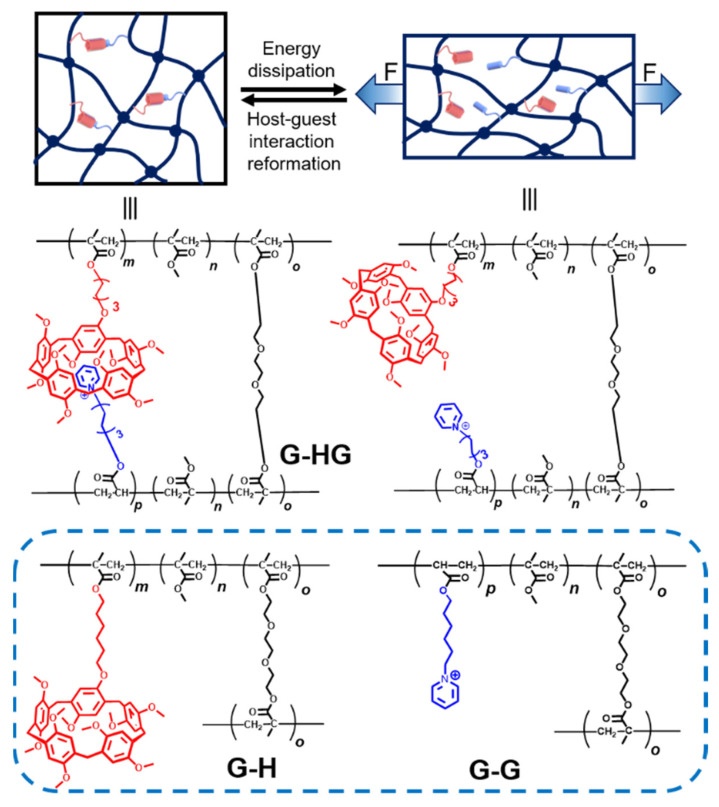
The energy dissipation mechanism of the polymer gel network bearing pillar[5]arene-based host–guest interactions and the chemical structures of **G-HG**, **G-H**, and **G-G** polymer gels.

Herein, we report a modified **G-HG** polymer gel via incorporation of pillar[5]arenes (P5) and pyridine cation (PC) side chains into a covalently crosslinked poly(methyl methacrylate) (PMMA) polymer network (Figure 1). The introduction of host–guest interactions will highly enhance the mechanical properties of the polymer gels. Upon mechanical loads, the host–guest complex can dissociate to dissipate vast quantities of energy, thereby dramatically enhancing the mechanical properties of the polymer gel. When the mechanical loads are withdrawn, the host–guest interactions will recover, thereby making the mechanical properties of the polymer gels reversible. To further prove the function of the host–guest interaction, we also designed two control polymer gels, including PMMA bearing solely P5 (**G-H**) and PMMA bearing solely PC (**G-G**) (Figure 1).

## 2. Results and Discussion

**Figure 2:**
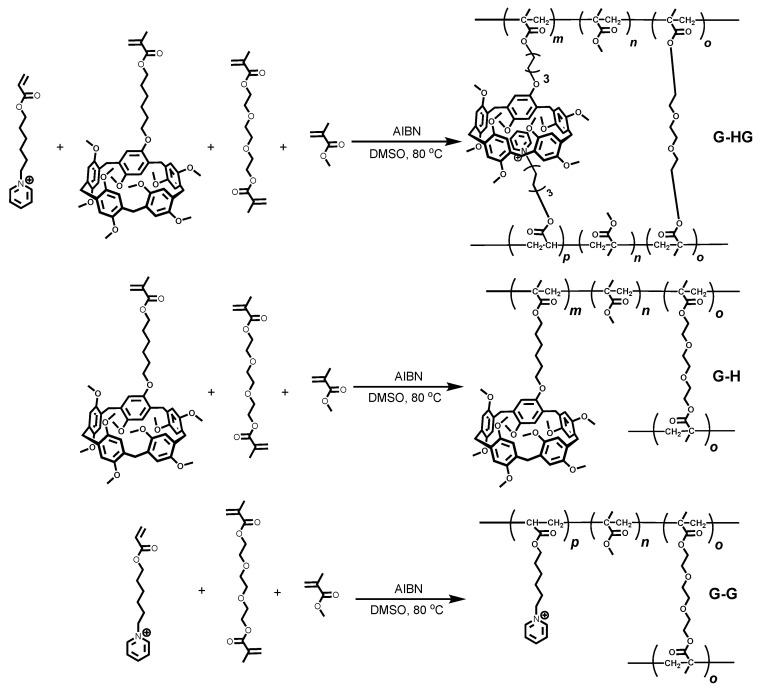
The synthetic routes used to obtain the **G-HG**, **G-H**, and **G-G** gels.

As shown in Figure 2, the model polymer gel (**G-HG**) was prepared via free radical copolymerization of methyl methacrylate (MMA), P5-modified MMA monomer, PC-modified MMA monomer, and covalent crosslinker poly (ethylene glycol) diacrylate (PEGDA). Due to the molecular recognition between P5 and PC, the **G-HG** network bears the host–guest interactions. As for the two control polymer gels, **G-H** and **G-G** were prepared via the copolymerization of MMA, modified MMA monomer (P5 or PC-modified MMA), and PEGDA. All the polymer gels were prepared in dimethyl sulfoxide (DMSO) under a nitrogen atmosphere, during which PEGDA was used as the covalent crosslinker. Characterized by attenuated total reflection-Fourier transform infrared (ATR-FTIR), the peak around 1723 cm^−1^ proved the presence of MMA units in the **G-G**, **G-H**, and **G-HG** polymer gels [64] (Figure S15). Additionally, the network structures of three polymer gels were evidenced by scanning electron microscopy (SEM, Figure S16), shown in Figure S16, consistent with the formation of crosslinked structures. The detailed synthesis (Scheme S1) and characterization of the monomers and polymer gels are shown below.

### 2.1. The Tensile Tests of the Gels

**Figure 3:**
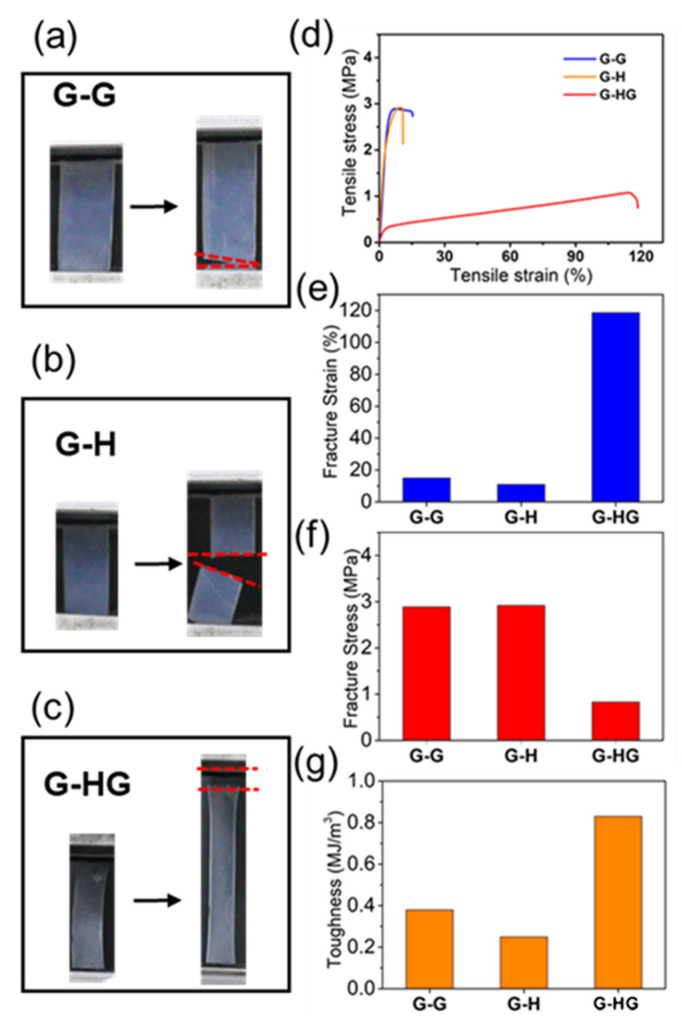
Photographs of the polymer gels of (**a**) **G-G**, (**b**) **G-H**, and (**c**) **G-HG** during the tensile tests. (**d**) Stress–strain curves, (**e**) fracture strain, (**f**) fracture stress, and (**g**) toughness of the **G-G**, **G-H**, and **G-HG** polymer gels.

The tensile tests were performed to evaluate the effect of the host–guest interactions on the mechanical properties in our system (Figure 3a–c, Movies S1–S3). A dramatic increase in final fracture strain was observed after the incorporation of the P5-based host–guest interactions (Figure 3d). **G-HG** exhibited an almost eight-fold final fracture strain, achieving a value near 118.6% (Figure 3e), while the highest value of **G-H** and **G-G** was only 15%. Compared to the other two control gels, **G-HG** achieved a fracture stress of 0.83 MPa (Figure 3f). Apart from the final fracture strain, the **G-HG** polymer gel also displayed an excellent toughness of 0.83 MJ/m^3^; in contrast, the values of **G-H** and **G-G** merely reached 0.25 MJ/m^3^ and 0.38 MJ/m^3^, respectively, exhibiting much lower toughness (Figure 3g). These increases observed in **G-HG** can be attributed to the effective energy dissipation mechanism due to the host–guest interactions between P5 and PC. The results reflected the remarkable influence of the incorporation of host–guest interactions on the gels’ mechanical performance.

### 2.2. The Rheological Experiment of the Gels

**Figure 4:**
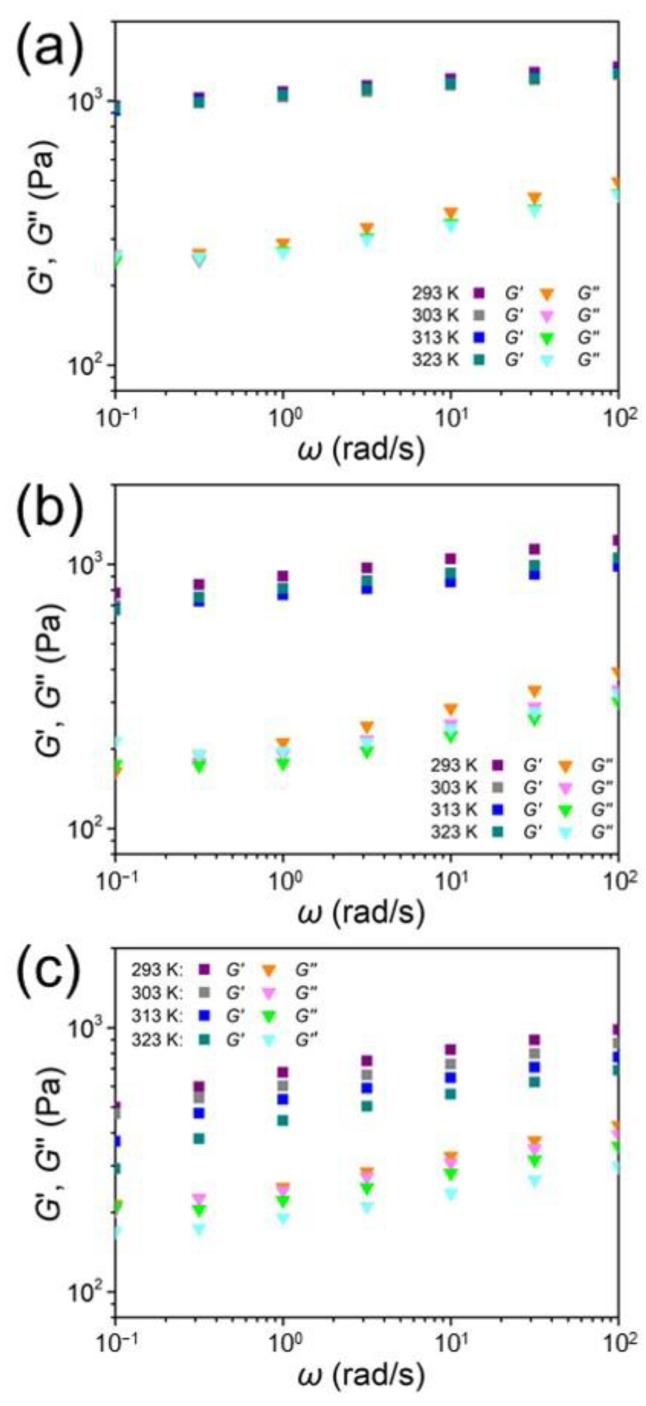
Storage (square) and loss (triangle) moduli versus frequency at different temperatures for the polymer gels (**a**) **G-G**, (**b**) **G-H**, and (**c**) **G-HG**.

To further determine the effect of the P5-based host–guest interactions in **G-HG** on the dynamic mechanical performance, we studied the rheological properties of the model polymer gels and the control polymer gels by determining their storage and loss moduli at different frequencies and temperatures. As shown in Figure 4, with the increase in temperature, the rheological experiment of gels **G-G** (Figure 4a) and **G-H** (Figure 4b) remained relatively constant at the same frequency. When the temperature rose from 293 K to 323 K, in contrast to both **G-G** and **G-H**, the storage and loss moduli of **G-HG** changed in a large range, showing a relatively higher temperature dependence. This can be ascribed to the reformation of the host–guest interactions in **G-HG** being temperature dependent [14]. Thus, given the host–guest interactions in the **G-HG** network, the **G-HG** polymer gel reflected a relatively higher sensitivity to temperature upon dynamic mechanical loading and unloading. Additionally, the loss moduli of **G-HG** of different temperatures showed similar values at low frequencies, while a noticeable difference was observed in its loss moduli at high frequencies (Figure 4c). Presumably, at low frequencies, the rate of the reformation of the host–guest interactions in our system was high enough to dissipate the energy efficiently, thereby reducing the interference of temperature in the loss moduli of **G-HG**. However, with the increase in frequency, in the case of a stronger mechanical loading at high frequencies, the rate of reformation of the host–guest interactions decreased dramatically; the effect of temperature gradually showed its dominance, leading to the higher difference of the loss moduli at different temperatures. This phenomenon was also reported in some systems containing hydrogen bonds and different kinds of host–guest interactions [14,15].

### 2.3. The Swelling Experiment of the Gels

**Figure 5:**
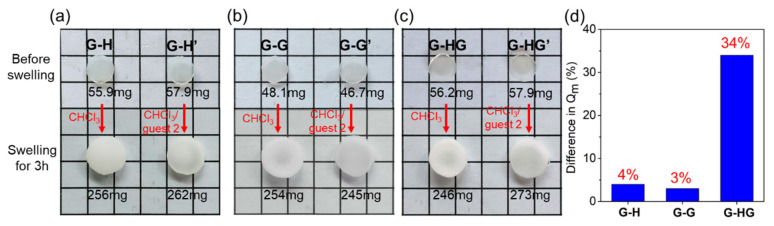
Photographs of polymer gels of (**a**) **G-H** and **G-H’**, (**b**) **G-G** and **G-G’**, and (**c**) **G-HG** and **G-HG’** before and after soaking in CHCl_3_ or 25 mM CHCl_3_/**guest 2** solution, respectively. The circular sheet samples of gels immersed in CHCl_3_ were called **G-H**, **G-G**, and **G-HG**, whereas those soaked in 25 mM **guest 2**/CHCl_3_ solution were labeled **G-H’**, **G-G’**, and **G-HG’**, respectively. The square of figure is 1 cm × 1 cm. (**d**) The difference in the mass swelling ratio of the two samples of **G-H**, **G-G**, and **G-HG** gels.

We next investigated changes in gel swelling behavior in the presence of a competing molecule, an imidazolium cation (**guest 2**). According to previous reports [54,65], imidazolium cation can form stronger interactions with pillar[5]arenes, thus disrupting existing host–guest complexes in the gels. As shown in Figure 5a–5c, two circular sheet samples of the **G-H**, **G-G**, and **G-HG** gels were immersed in CHCl_3_ or 25 mM CHCl_3_/**guest 2** solution, respectively. The gels reached swelling equilibrium after 3 h. The mass swelling ratio of each gel was calculated by the following formula:

*Q*_m_ = (*m*_s_ − *m*)/*m* (1)

where *Q*_m_ is the mass swelling ratio (%) of the gel, and *m* and *m_s_* represent the mass of the gel before and after swelling. The *Q*_m_ values of the two samples of each gel are shown in Table S1. Then, we compared the differences in the mass swelling ratios of the two samples of each gel, as shown in Figure 5d. The difference in the mass swelling ratios of the two samples of **G-H**, **G-G**, and **G-HG** gels were 4%, 3%, and 34%, respectively. It was clearly found that the difference in the swelling ratios of the two **G-HG** gel samples was much larger than that of the other gel samples. These obtained results can be ascribed to the destruction of the existing host–guest complex of the **G-HG** gel, followed by the involved non-covalent crosslinks vanishing, leading to the crosslink drop of the gel. The above reasons led the **G-HG** gel to swell more easily after soaking in CHCl_3_/**guest 2** solution, causing a higher mass swelling ratio.

## 3. Conclusions

In summary, we report here a polymer gel modified by the incorporation of the host–guest interactions between the pillar[5]arenes and pyridine cation to construct a dual crosslinked polymer network. The incorporated host–guest interactions can be used as sacrificial non-covalent bonds that can dissociate upon mechanical loads to dissipate vast quantities of energy, thereby enhancing the mechanical properties dramatically. Relative to the control polymer gels without bearing the host–guest interactions, the model polymer gel exhibited an almost eight-fold increase in final fracture strain, achieving a value near 118.6%. The effect of host–guest interactions on the gels’ mechanical performance was further determined by measuring their rheological properties and by performing swelling experiments. The dual crosslinked polymer gels with extraordinary mechanical performance present a promising strategy, affording more choices of polymer gels for numerous applications, such as tissue engineering, biomedicine, and sensing, etc.

## 4. Materials and Methods

### 4.1. Materials and Instruments

Poly (ethylene glycol) diacrylate (PEGDA) and BF_3_·Et_2_O were obtained from Macklin (Shanghai, China). 1,6-dibromohexane and paraformaldehyde were purchased from Aladdin (Shanghai, China). 4-Methoxyphenol was obtained from Leyan (Shanghai, China). Pyridine, azobisisobutyronitrile (AIBN), extra dry dimethyl formamide (DMF), extra dry dimethyl sulfoxide (DMSO), extra dry acetonitrile, and extra dry dichloromethane (DCM) were procured from Energy Chemical (Shanghai, China). Potassium carbonate (K_2_CO_3_), potassium iodide (KI), methacrylic acid, and toluene were purchased from SINOPHARM (Shanghai, China). Chloroform was obtained from KeShi (Chengdu, China). Trifluoroacetic acid (TFA) was procured from Aike Reagent (Chengdu, China). All reagents were purchased from commercial suppliers and used without further purification. Solvents were either employed as purchased or purified by standard methods prior to use. Compound **3** was prepared according to the procedure described in the literature [54]. ^1^H NMR and ^13^C NMR spectra were performed with a Bruker Advance 400 MHz spectrometer. High-resolution electrospray ionization mass spectra (ESI-MS) were recorded using a Bruker microOTOF II. The rheological properties of the gels were measured using a rheometer MCR 302 (Anton Paar, Austria). The tensile tests of the gels were investigated using an electronic universal testing machine (CMT4104, Shenzhen San Testing Machine Co., Shenzhen, China) with a tensile rate of 7 mm/min. Attenuated total reflection-Fourier transform infrared (ATR-FTIR) spectroscopy was recorded on a Bruker spectrometer (Vertex 70, Karlsruhe, Germany). Scanning electron microscope (SEM) images of freeze-dried gels were obtained using a Hitachi SU8010 instrument, Hitachi, Tokyo, Japan.

### 4.2. Synthesis and Characterization of Compounds ***3*** and ***4*** and ***Guest 2***

#### 4.2.1. Synthesis and Characterization of Compound **3**

4-Methoxyphenol (12.4 g, 0.100 mol) and K_2_CO_3_ (22.0 g, 0.160 mol) were dispersed in acetonitrile (200 mL) and the mixture was stirred at room temperature for 30 min. Then, KI (0.200 g, 10.0 mmol) and excess 1,6-dibromohexane (18.5 mL, 0.120 mol) were added to the solution. The mixture was added to reflux condenser and reacted for 16 h. The solution was concentrated under vacuum and subjected to silica gel chromatography (petroleum ether (PE)/ethyl acetate (EA), 10:1, *v*/*v*) to give the product **1** (17.9 g, yield: 90%). ^1^H NMR (Figure S1) (400 MHz, CDCl_3_, 298 K) *δ* (ppm): 6.83 (s, 4 H), 3.91 (t, *J* = 6.4 Hz, 2 H), 3.77 (s, 3 H), 3.42 (t, *J* = 6.8 Hz, 2 H), 1.89 (t, *J* = 10.2 Hz, 2 H), 1.78 (q, *J* = 6.7 Hz, 2 H), 1.52 − 1.46 (m, 4 H). ^13^C NMR (Figure S2) (100 MHz, CDCl_3_, 298 K) *δ* (ppm): 153.84, 153.32, 115.54, 114.75, 77.16, 68.49, 55.85, 33.94, 32.82, 29.33, 28.06, 25.43.

Compound **1** (1.15 g, 4.00 mmol), 1,4-dimethoxybenzene (2.75 g, 20 mmol), paraformaldehyde (2.52 g, 84.0 mmol), and DCM (180 mL) were added into a flask under an ice-water bath and stirred for 30 min. Then, BF_3_·Et_2_O (3.60 mL) was added into the flask. After the color of solution changed from white to light yellow to olivine to dark green (ca. 40 min), water (300 mL) was poured into solution to quench the reaction. The pure compound **2** was obtained as white power (556 mg, yield: 15%) over silicone gel column chromatography (PE:DCM:EA = 90:30:1). ^1^H NMR (Figure S3) (400 MHz, CDCl_3_, 298 K) *δ* (ppm): 7.02 − 6.78 (m, 10 H), 3.87 − 3.67 (m, 41 H), 1.30 − 1.25 (m, 4 H), 0.89 − 0.83 (m, 4 H). ^13^C NMR (Figure S4) (100 MHz, CDCl_3_, 298 K) *δ* (ppm): 151.21, 150.86, 150.42, 150.31, 149.64, 128.34, 128.21, 128.09, 128.05, 114.09, 113.40, 113.07, 69.00, 55.85, 55.39, 55.29, 33.16, 30.76, 29.72, 29.24, 29.03, 27.78, 24.08. HRMS (ESI^+^) (Figure S5) Calcd for C_50_H_59_BrO_10_ [M + Na]^+^: 923.3169, found: 923.3172.

Methacrylic acid (980 mg, 11.4 mmol) and potassium carbonate (157 mg, 1.14 mmol) were added in dry dimethyl formamide (20 mL) under stirring at room temperature for 0.5 h, then compound **2** (1.70 g, 1.90 mmol) was added. The mixture was stirred at room temperature for 24 h. After the reaction was completed, the resulting mixture was evaporating of the solvent under reduced pressure and further purification was carried out by column chromatography using PE/EA as an eluent to afford 500 mg of product **3** as a white solid. Yield: 29%.^1^H NMR (Figure S6) (400 MHz, CDCl_3_, 298 K) *δ* (ppm): 6.99 − 6.77 (m, 10 H), 6.12 (s, 1 H), 5.57 (s, 1 H), 4.16 (s, 2 H), 3.85 − 3.49(m, 39 H), 1.97 (s, 3 H), 1.81 − 1.44 (m, 8 H). ^13^C NMR (Figure S7) (100 MHz, CDCl_3_, 298 K) *δ* (ppm): 167.62, 150.90, 150.21, 136.66, 128.46, 128.39, 128.33, 128.29, 125.36, 115.15, 114.28, 64.82, 55.90, 29.87, 29.82, 29.57, 29.20, 28.77, 26.18, 26.03, 22.95, 18.49. HRMS (ESI^+^) (Figure S8) Calcd for C_54_H_64_O_12_ [M + Na]^+^: 927.4296, found: 927.4263.

#### 4.2.2. Synthesis and Characterization of Compound **4**

A solution of 6-bromohexyl acrylate (3.06 g, 13.0 mmol) and pyridine (5.14 g, 65 mmol) in toluene (45 mL) was refluxed at 80 °C for 24 h. The solution was then concentrated, dissolved in 3 mL ethanol, precipitated in 40 mL diethyl ether, and washed with petroleum ether to obtain a pale yellow oil **4** (1.60 g, 53%). ^1^H NMR (Figure S9) (400 MHz, D_2_O, 298 K) *δ* (ppm): 8.85 (d, *J* = 5.7 Hz, 2 H), 8.54 (t, *J* = 7.8 Hz, 1 H), 8.07 (t, *J* = 6.9 Hz, 2 H), 6.39 (d, *J* = 18.2 Hz, 1 H), 6.17 (dd, *J* = 17.3, 10.5 Hz, 1 H), 5.95 (d, *J* = 11.4 Hz, 1 H), 4.62 (t, *J* = 7.3 Hz, 2 H), 4.16 (t, *J* = 6.5 Hz, 2 H), 2.03 (p, *J* = 7.2 Hz, 2 H), 1.68 (p, *J* = 6.6 Hz, 2 H), 1.46 − 1.33 (m, 4 H). ^13^C NMR (Figure S10) (100 MHz, D_2_O, 298 K) *δ* (ppm): 168.80, 145.59, 144.21, 132.22, 128.27, 127.76, 65.35, 61.85, 30.43, 27.51, 24.87, 24.62. HRMS (ESI^+^) (Figure S11) Calcd for C_14_H_20_NO_2_^+^ [M]^+^: 234.1489, found: 234.1496.

### 4.3. Synthesis and Characterization of ***Guest 2***

The **guest 2** was synthesized referring to the related literature [59]. 1-Butylimidazole (2.0 g, 16 mmol) and trifluoroacetic acid were dissolved in chloroform (20 mL), which was stirred at room temperature for 30 min. After removing the solvents under reduced pressure, we obtained **guest 2** as a colorless oil (3.82 g, 100%). ^1^H NMR (Figure S12) (400 MHz, CDCl_3_, 298 K) *δ* (ppm): 8.79 (s, 1 H), 7.44 (s, 1 H), 7.18 (s, 1 H), 4.18 (t, *J* = 8.0 Hz, 2 H), 1.92 − 1.84 (m, 2 H), 1.43 − 1.33 (m, 2 H), 0.98 (t, *J* = 8.0 Hz, 3 H). ^13^C NMR (Figure S13) (100 MHz, CDCl_3_, 298 K) *δ* (ppm): 135.15, 121.02, 120.99, 49.84, 32.25, 19.63, 13.43. HRMS (ESI^+^) (Figure S14) Calcd for C_7_H_13_N_2_^+^ [M]^+^: 125.1073, found: 125.1114.

### 4.4. Synthesis of ***G-HG***, ***G-H***, and ***G-G*** Gels

#### 4.4.1. Synthesis of Gel **G-HG**

Gel **G-HG** was prepared from compounds **3** and **4**, poly (ethylene glycol) diacrylate (PEGDA), and methyl methacrylate by free radical polymerization. A mixture of compound 3 (316.4 mg, 0.350 mmol), compound **4** (82.0 mg, 0.350 mmol), poly (ethylene glycol) diacrylate (PEGDA) (129 mg, 0.450 mmol), and methyl methacrylate (700 mg, 7.00 mmol) in 3.50 mL of DMSO was stirred at room temperature. A stream of nitrogen was bubbled through the reaction mixture for 30 min. AIBN (12.3 mg, 0.0750 mmol) was then added in one portion and the mixture was stirred for 10 min, sealed with a rubber septum and heated to 80 °C for 8 h, then gel **G-HG** was obtained.

#### 4.4.2. Synthesis of Gel **G-H**

Gel **G-H** was prepared from compound **3**, poly (ethylene glycol) diacrylate (PEGDA), and methyl methacrylate by free radical polymerization. A mixture of compound **3** (316.4 mg, 0.350 mmol), poly (ethylene glycol) diacrylate (PEGDA) (129 mg, 0.450 mmol), and methyl methacrylate (700 mg, 7.00 mmol) in 3.50 mL of DMSO was stirred at room temperature. A stream of nitrogen was bubbled through the reaction mixture for 30 min. AIBN (12.3 mg, 0.0750 mmol) was then added in one portion and the mixture was stirred for 10 min, sealed with a rubber septum, and heated to 80 °C for 8 h, then gel **G-H** was obtained.

#### 4.4.3. Synthesis of Gel **G-G**

Gel **G-G** was prepared from compound **4**, poly (ethylene glycol) diacrylate (PEGDA) and methyl methacrylate by free radical polymerization. A mixture of compound **4** (82.0 mg, 0.350 mmol), poly (ethylene glycol) diacrylate (PEGDA) (129 mg, 0.450 mmol), and methyl methacrylate (700 mg, 7.00 mmol) in 3.50 mL of DMSO was stirred at room temperature. A stream of nitrogen was bubbled through the reaction mixture for 30 min. AIBN (12.3 mg, 0.0750 mmol) was then added in one portion and the mixture was stirred for 10 min, sealed with a rubber septum, and heated to 80 °C for 8 h, then gel **G-G** was obtained.

## Data Availability

Not applicable.

## Supplementary Materials

The following supporting information can be downloaded at: https://www.mdpi.com/article/10.3390/gels8080475/s1, Scheme S1: Synthetic routes of compounds **3** and **4** and **guest 2**. Figure S1: ^1^H NMR spectrum (CDCl_3_, 400 MHz, 298 K) of compound **1**. Figure S2: ^13^C NMR spectrum (CDCl_3_, 100 MHz, 298 K) of compound **1**. Figure S3: ^1^H NMR spectrum (CDCl_3_, 400 MHz, 298 K) of compound **2**. Figure S4: ^13^C NMR spectrum (CDCl_3_, 100 MHz, 298 K) of compound **2**. Figure S5: HR-ESI^+^-MS spectrum of compound **2**. Figure S6: ^1^H NMR spectrum (CDCl_3_, 400 MHz, 298 K) of compound **3**. Figure S7: ^13^C NMR spectrum (CDCl_3_, 100 MHz, 298 K) of compound **3**. Figure S8: HR-ESI^+^-MS spectrum of compound **3**. Figure S9: ^1^H NMR spectrum (D_2_O, 400 MHz, 298 K) of compound **4**. Figure S10: ^13^C NMR spectrum (D_2_O, 100 MHz, 298 K) of compound **4**. Figure S11: HR-ESI^+^-MS spectrum of compound **4**. Figure S12: ^1^H NMR spectrum (CDCl_3_, 400 MHz, 298 K) of **guest 2**. Figure S13: ^13^C NMR spectrum (CDCl_3_, 100 MHz, 298 K) of **guest 2**. Figure S14: HR-ESI^+^-MS spectra of **guest 2**. Figure S15: ATR-FTIR spectra of polymer gels (a) **G-G**, (b) **G-H**, and (c) **G-HG**. Figure S16: SEM images of polymer gels (a) **G-G**, (b) **G-H**, and (c) **G-HG**. Table S1: The *Q*_m_ values of the two samples of each gel. Movie S1; Movie S2; Movie S3.
