# Supplementary material for: Enhancing Mechanical Performance of a Polymer Material by Incorporating Pillar[5]arene-Based Host–Guest Interactions
Source: Gels. 2022 Jul 28;8(8):475. doi: 10.3390/gels8080475 (PMC9407059; doi:10.3390/gels8080475)
Supplement: Supplementary file 1 [file gels-08-00475-s001.zip › gels-1821583-supplementary.pdf]

gels

MDPI

Communication

Enhancing Mechanical Performance of a Polymer Material by Incorporating Pillar[5]arene-Based Host–Guest Interactions

Chengdi Huang, Hanwei Zhang, Ziqing Hu, Youping Zhang and Xiaofan Ji *

School of Chemistry and Chemical Engineering, Huazhong University of Science and Technology, Wuhan 430074, China; chengdihuang@hust.edu.cn (C.H.); zhanghanwei@hust.edu.cn (H.Z.); huziqingbjt@163.com (Z.H.); u201810425@hust.edu.cn (Y.Z.)

* Correspondence: xiaofanji@hust.edu.cn

Supplementary Materials

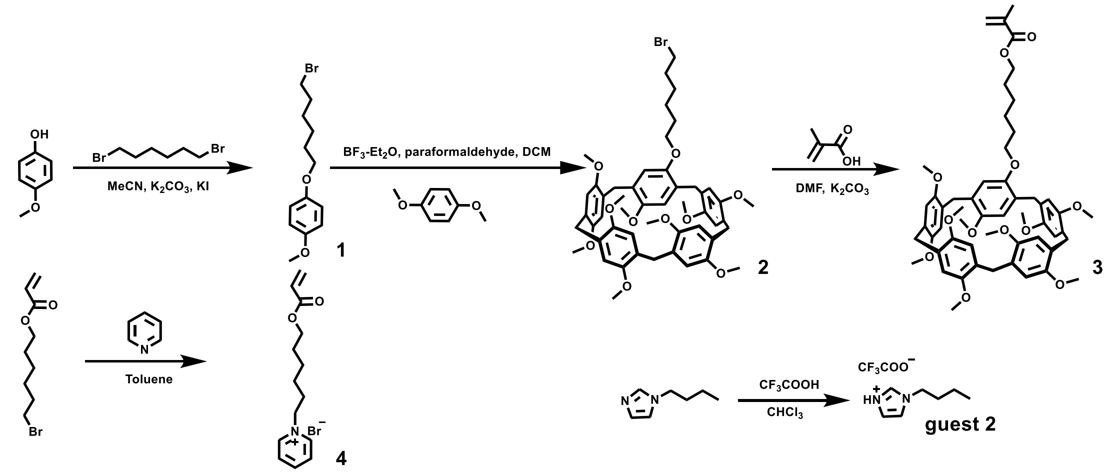

Scheme S1. Synthetic routes of compound **3, 4** and **guest 2**.

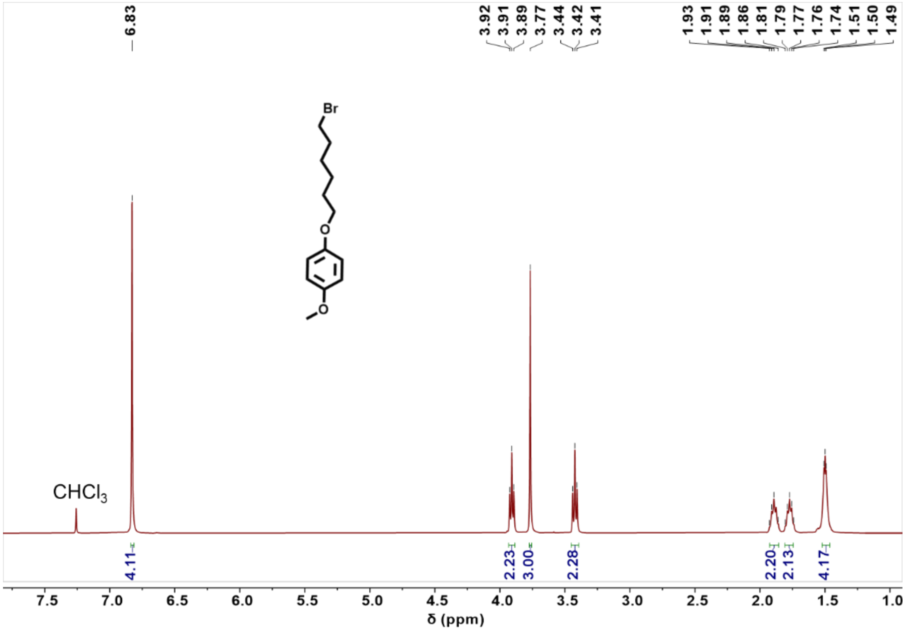

Figure S1. ^{1}H NMR spectrum ($CDCl_3$, 400 MHz, 298 K) of compound **1**.

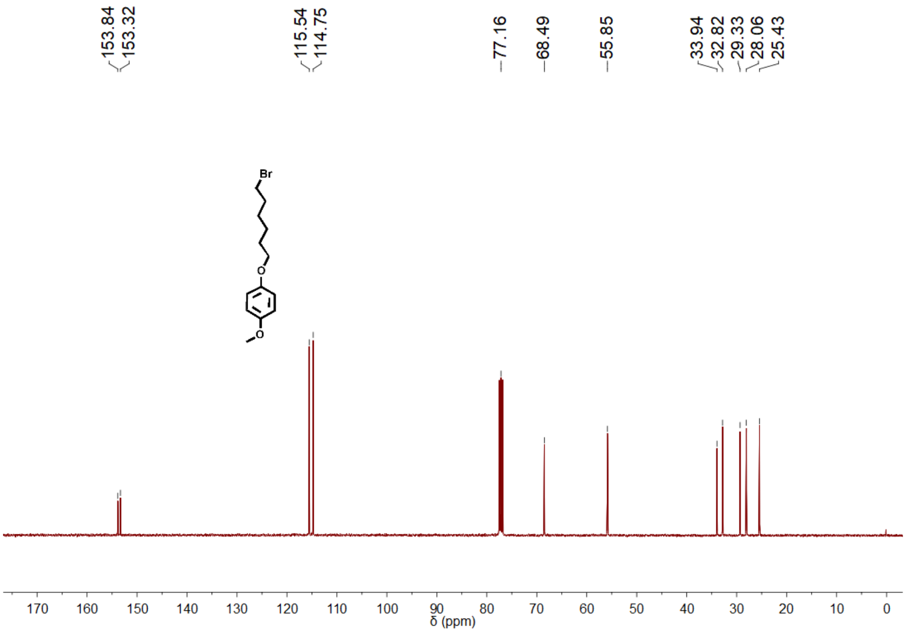

Figure S2. ^{13}C NMR spectrum ($CDCl_3$, 100 MHz, 298 K) of compound **1**.

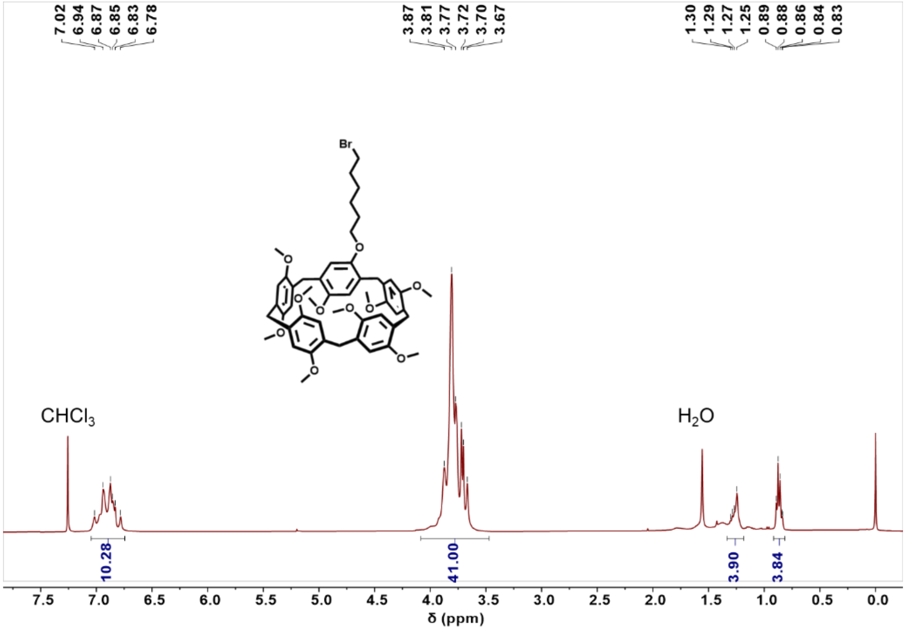

Figure S3. ^{1}H NMR spectrum ($CDCl_3$, 400 MHz, 298 K) of compound **2**.

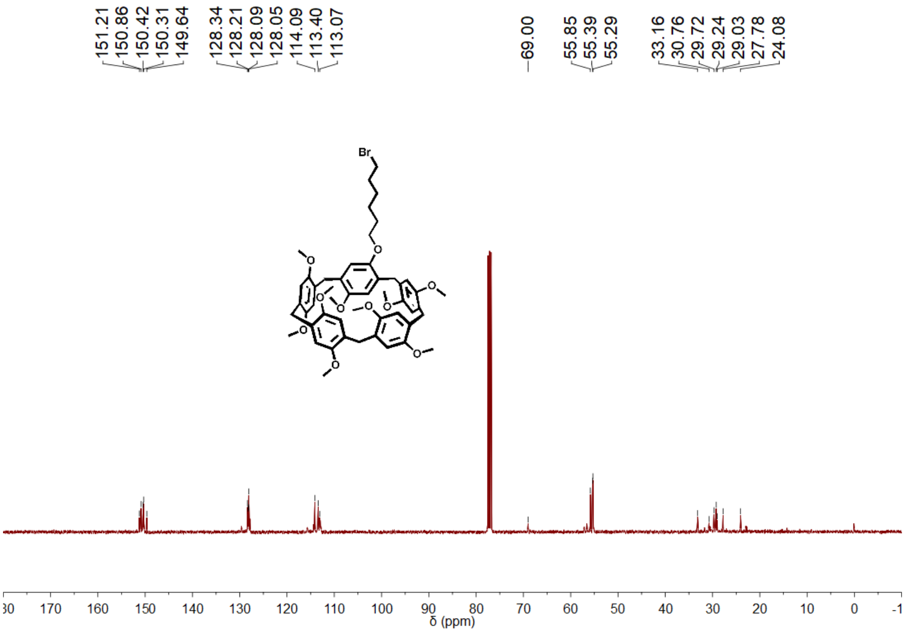

Figure S4. ^{13}C NMR spectrum ($CDCl_3$, 100 MHz, 298 K) of compound **2**.

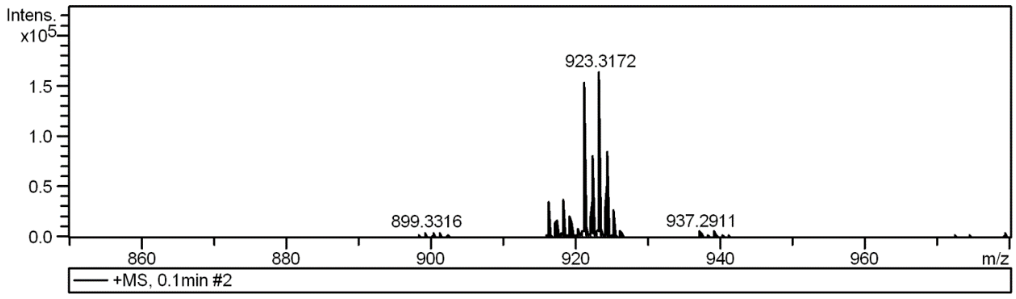

Figure S5. HR-ESI+-MS spectrum of compound **2**.

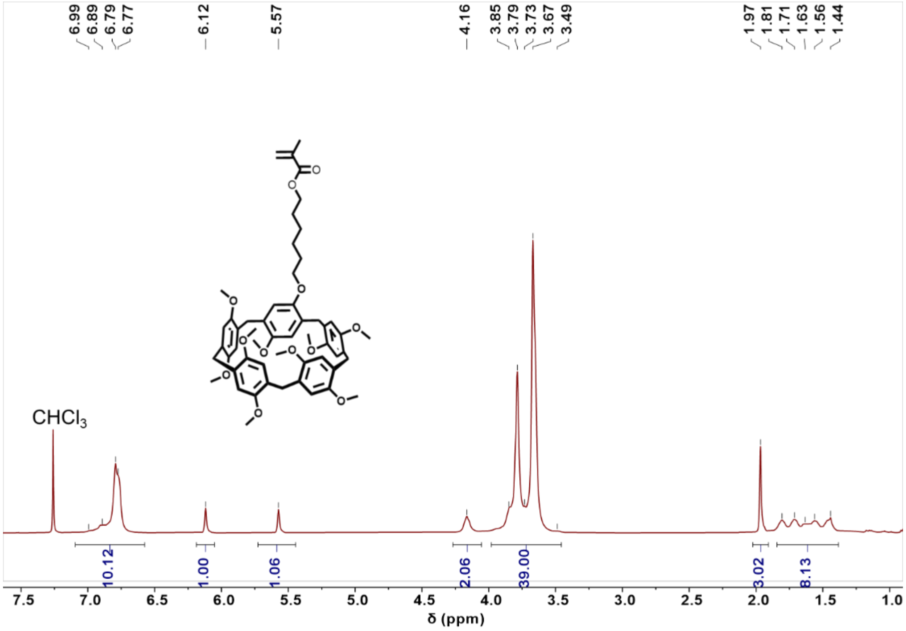

Figure S6. ^{1}H NMR spectrum ($CDCl_3$, 400 MHz, 298 K) of compound **3**.

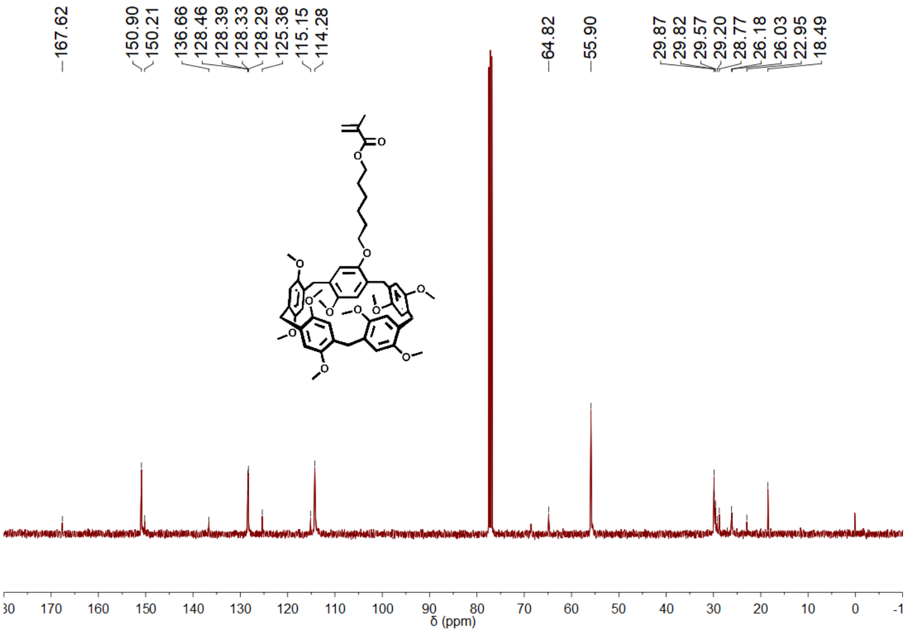

Figure S7. ^{13}C NMR spectrum ($CDCl_3$, 100 MHz, 298 K) of compound **3**.

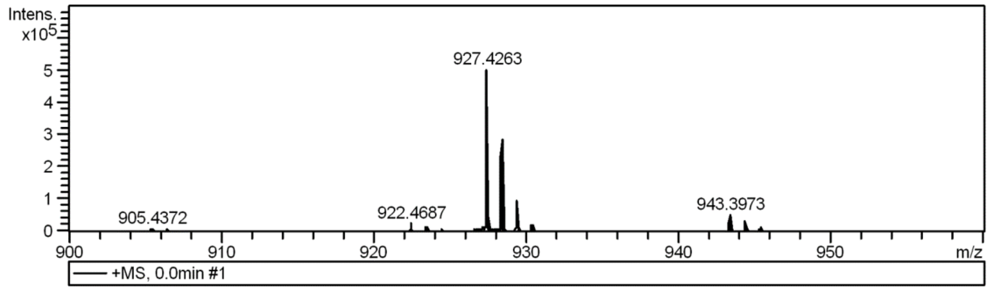

Figure S8. HR-ESI$^+$-MS spectrum of compound **3**.

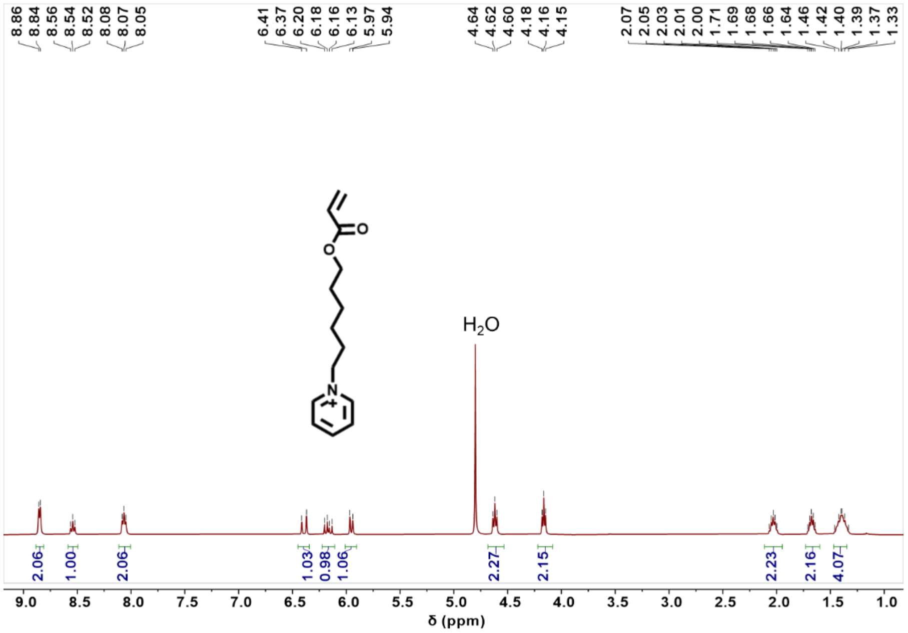

Figure S9. ^{1}H NMR spectrum (D_2O, 400 MHz, 298 K) of compound **4**.

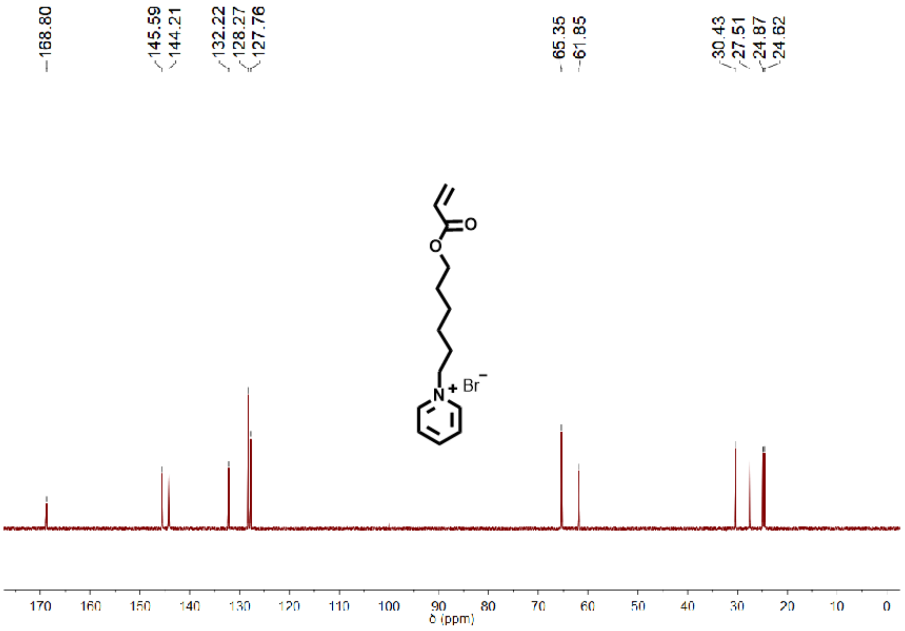

Figure S10. ^{13}C NMR spectrum (D_2O, 100 MHz, 298 K) of compound **4**.

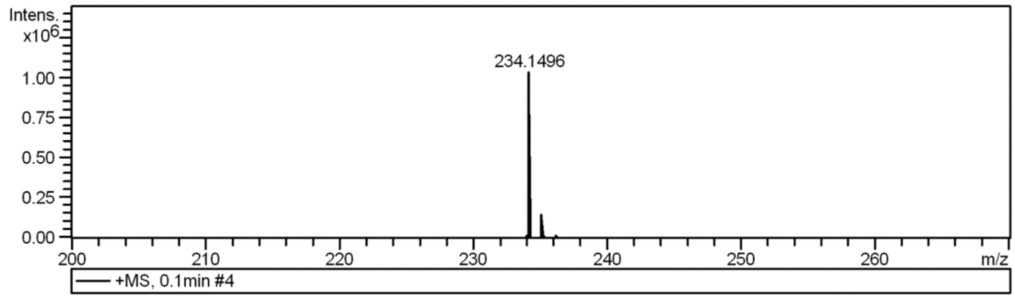

Figure S11. HR-ESI[+]-MS spectrum of compound **4**.

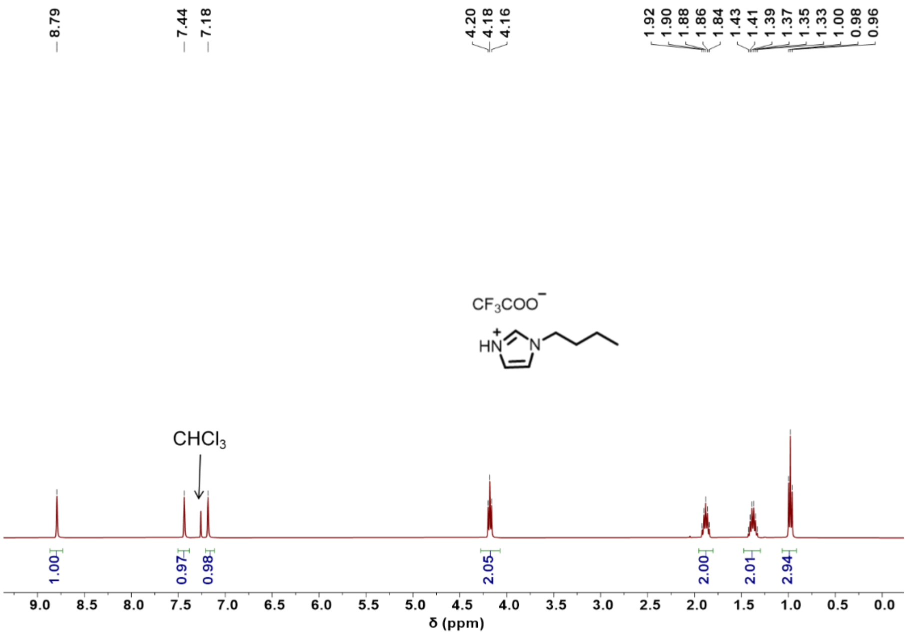

Figure S12. ^{1}H NMR spectrum ($CDCl_3$, 400 MHz, 298 K) of **guest 2**.

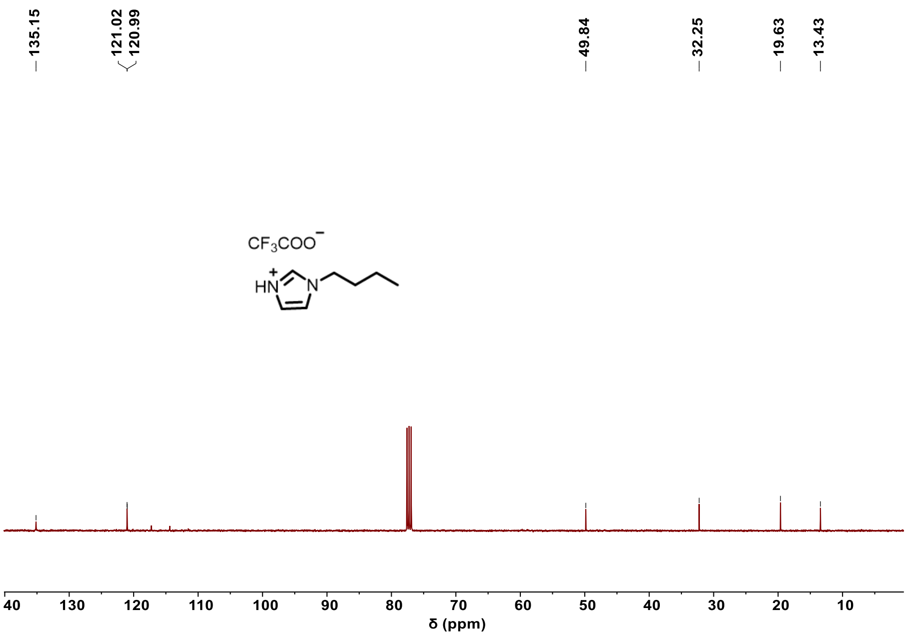

Figure S13. ^{13}C NMR spectrum ($CDCl_3$, 100 MHz, 298 K) of **guest 2**.

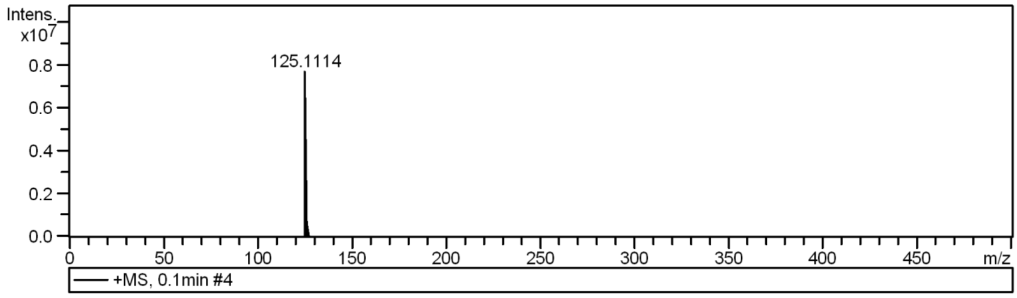

Figure S14. HR-ESI$^+$-MS spectrum of compound **guest 2**.

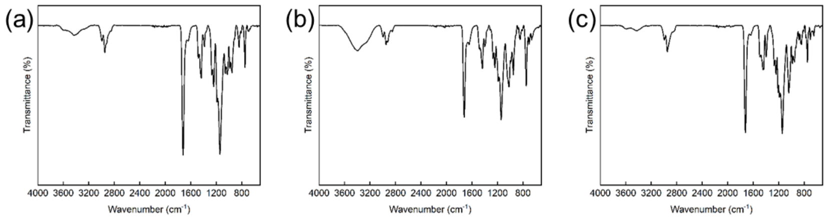

Figure S15. ATR-FTIR spectra of polymer gels (a) **G-G**, (b) **G-H**, and (c) **G-HG**.

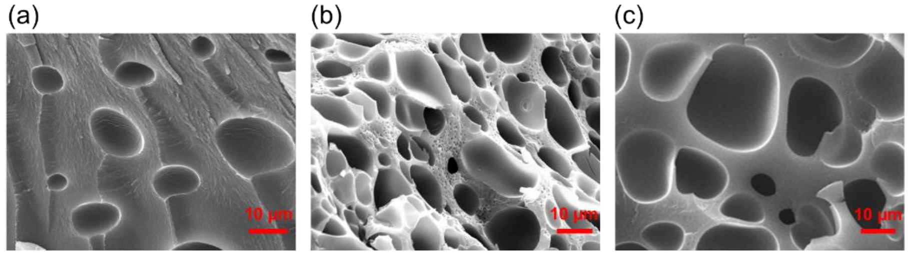

Figure S16. SEM images of polymer gels (a) **G-G**, (b) **G-H**, and (c) **G-HG**.

Table S1. The Q_m values of the two samples of each gel*.

Gels	G-H	G-H′	G-G	G-G′	G-HG	G-HG′
Q_m	358%	354%	428%	425%	338%	372%

* The circular sheet samples of gels immersed in $CHCl_3$ were called **G-H**, **G-G** and **G-HG**,whereas they soaked in 25 mM $CHCl_3$/ **guest 2** solution were labeled **G-H′**, **G-G′** and **G-HG′,** respectively.
